# Characterisation of a novel poly (ether ether ketone)/calcium sulphate composite for bone augmentation

**DOI:** 10.1186/s40824-017-0093-7

**Published:** 2017-05-19

**Authors:** Erik A. B. Hughes, Liam M. Grover

**Affiliations:** School of Chemical Engineering, University of Birmingham, Edgbaston, B15 2TT UK

**Keywords:** Bone graft, Biomaterial, Calcium sulphate, PEEK

## Abstract

**Background:**

Calcium sulphate (CS) has been used in bone grafting since the 1800s. It has not replaced autograft as the gold standard, however, since its dissolution occurs rapidly in bodily fluids, meaning that the material cannot support long-term bone growth. Here, the polymer poly (ether ether ketone) (PEEK) was used to slow dissolution in in vitro physiological environments and augment the mechanical properties of the material.

**Methods:**

PEEK/CS specimens were fabricated by combining powders of PEEK and CS with water, resulting in a hardening paste. To enhance physical interactions between phases, cylindrical specimens were heat-treated to melt and fuse the PEEK. Following analysis of physical and chemical interactions by SEM and FT-IR respectively, dynamic ageing in PBS and compression testing was undertaken to measure how the PEEK influenced the mechanical properties of the final parts. Changes in structure and chemistry were determined using helium pycnometry, SEM and analysis of powder XRD patterns.

**Results:**

Powders of PEEK and CS hemihydrate (CSH) (CaSO_4_.0.5H_2_O) were combined with PEEK at 0 wt%, 2.5 wt%, 20 wt%, 40 wt% and 80 wt% and at a P:L ratio of 0.85 g/mL. The subsequently hardened structures were heat-treated, which initiated the melting of PEEK and dehydration of CSD (CaSO_4_.2H_2_O) to the CS anhydrite (CSA) (CaSO_4_) phase, which changed colour and apparent volume. FT-IR and SEM analysis revealed heat treatment of PEEK/CS specimens facilitated both physical and chemical interactions between phases. Over a period of 21 days of ageing in PBS, the hydration of CS was determined by XRD and improved specimen longevity at all levels of PEEK wt% loading was measured compared with the control. Importantly, increasing PEEK wt% loading resulted in a marked increase in the mechanical properties of PEEK/CS specimens in terms of both compressive strength and modulus.

**Conclusions:**

Reinforcement of CS with PEEK significantly enhanced in vitro dissolution resistance, in addition to enhancing mechanical properties. This composite therefore has significant future potential as a bone graft replacement.

**Electronic supplementary material:**

The online version of this article (doi:10.1186/s40824-017-0093-7) contains supplementary material, which is available to authorized users.

## Background

Bone graft materials should support the attachment and proliferation of osteoblast cells, and facilitate the deposition of new hard tissue in bone defects [1]. Ideally, these materials allow for the conduction of new mineral deposition and demonstrate good integration with adjacent bony tissues [2]. Increasingly, synthetic minerals are chosen over bone harvested from patient’s own body. This is despite autologous bone graft being considered the “gold standard” graft material, due to the low risk of immunological rejection associated with its use [3, 4]. The reasons for alternative bone graft selection are that the volume of autologous bone is limited and requires additional surgery to obtain, which poses further risk to a patient in terms of contracting an infection, blood loss and experiencing unnecessary discomfort thereafter [4]. In addition, allograft tissue suffers drawbacks in terms of disease transmission and possible rejection [4].

Synthetic bioceramic materials, particularly calcium (Ca^2+^) salts of phosphate (PO_4_^3−^), pyrophosphate (P_2_O_7_^4−^), sulphate (SO_4_^2−^) and silicate (SiO_4_^4−^) are proven examples of minerals that are able to regenerate areas of removed or diseased hard tissue [5–11]. Moreover, these materials can be employed directly and are available in a variety of forms that includes granules and injectable cementitious pastes, allowing surgeons to select the most appropriate product on a case-by-case basis.

The first ceramic material in widespread use for skeletal regeneration was calcium sulphate (CS). The ability of CS to be set in situ or applied directed as granules means that the material can be delivered in a number of ways [12, 13]. CS hemihydrate (CSH) (CaSO_4_.0.5H_2_O) and CS anhydrite (CSA) (CaSO_4_) forms cement when mixed with water, which hardens to form CS dihydrate (CSD) (CaSO_4_.2H_2_O) (Equation 1 and 2). The interaction between phases is reversible, as the water of crystallization can be removed by heating CSD (CaSO_4_.2H_2_O) (Equation 3 and 4). CS was first used at the end of the 19^th^ century to provide a fully resorbable osteoconductive scaffold capable of facilitating new bone formation in the diseased tissue of human tuberculosis sufferers [9]. CS is able to facilitate cavity healing incurred from curettage of bone cysts, as well as being used successfully to augment osteoportic bone to allow for mechanical fixation of pedicle screws [10, 11, 14].

1 $$ {\mathrm{CaSO}}_4.\mathrm{0.5}{\mathrm{H}}_2\mathrm{O} + 1.5{\mathrm{H}}_2\mathrm{O}\to {\mathrm{CaSO}}_4.2{\mathrm{H}}_2\mathrm{O} + \mathrm{heat} $$

2 $$ {\mathrm{CaSO}}_4 + 2{\mathrm{H}}_2\mathrm{O}\to {\mathrm{CaSO}}_4.2{\mathrm{H}}_2\mathrm{O} + \mathrm{heat} $$

3 $$ {\mathrm{CaSO}}_4.2{\mathrm{H}}_2\mathrm{O} + \mathrm{heat}\to {\mathrm{CaSO}}_4.\mathrm{0.5}{\mathrm{H}}_2\mathrm{O} + 1.5{\mathrm{H}}_2\mathrm{O} $$

4 $$ {\mathrm{CaSO}}_4.2{\mathrm{H}}_2\mathrm{O} + \mathrm{heat}\to {\mathrm{CaSO}}_4 + 2{\mathrm{H}}_2\mathrm{O} $$

Whilst resorbable graft materials are desirable, CS has been limited in its application since it undergoes rapid dissolution by hydrolytically driven degradation when placed in the body [15–17]. In contrast, biominerals such as hydroxyapatite (Ca_5_(PO_4_)_3_OH) (HA) are considerably less soluble in physiological conditions, offering a more stable network to support bone formation [18, 19]. Given that newly forming bone may take several weeks to fill a defect, these more stable calcium phosphate ceramics are favoured over CS for defect augmentation [6, 13].

Strategies to improve the degradation behavior of CS include combination with less soluble mineral phases and additives to from composites that possess enhanced properties. In combination with β-tricalcium phosphate (β-TCP) CS still undergoes dissolution but the calcium phosphate mineral is able to remain at the implant site for a longer period [20]. Composites containing CS can be modified to adjust degradation rate whilst dampening the production of acidic dissolution products [17, 21]. The addition of other phases can also augment the mechanical properties of the graft materials, with previous work showing that HA addition to CS may significantly enhance mechanical properties [22]. Attempts to combine CS with carboxymethylcellulose and hyaluronan improved mechanical properties of the material but at a cost of faster dissolution [23].

**Fig. 1:**
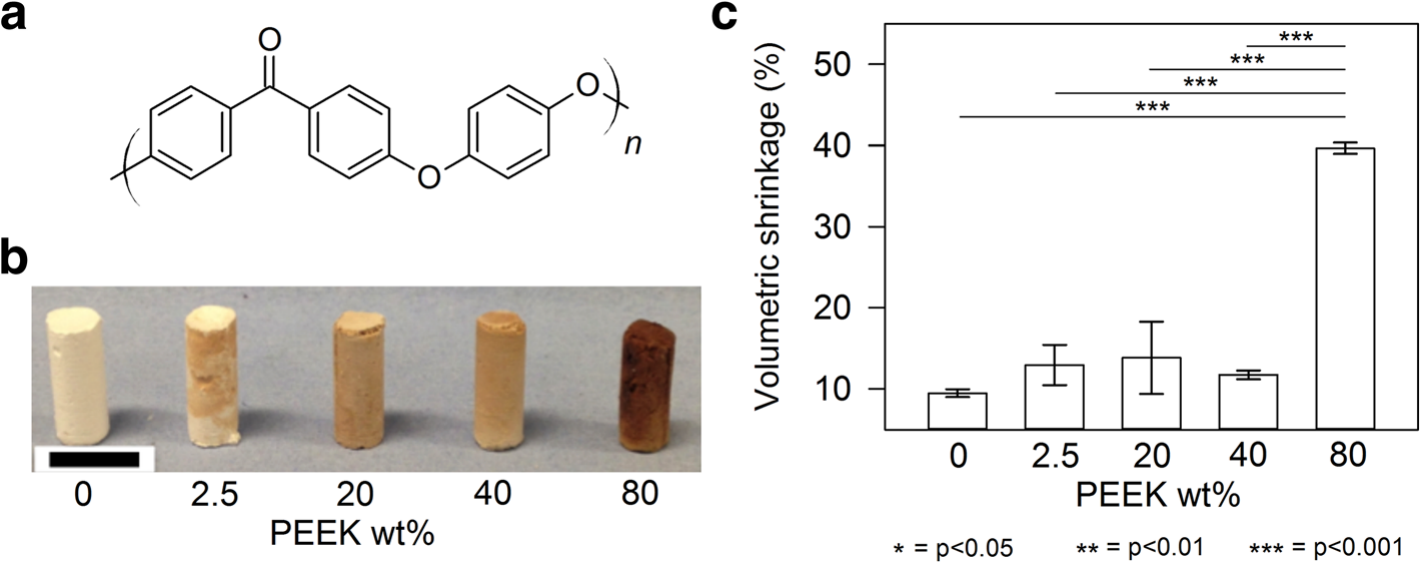
(**a**) Chemical structure of the poly(ether ether ketone) repeat monomer unit. (**b**) PEEK/CS specimens post heat treatment (Scale bar is equal to 10 mm). (**c**) Volumetric shrinkage of PEEK/CS specimens due to heat treatment. Error bars represent standard deviation (*n* = 3) and lines above data columns represent significant differences between groups based on PEEK wt% loading as found by post-hoc Turkey HSD tests following one-way ANOVA analysis

It was postulated that combination of CS with an engineering polymer phase could allow for tunable hydrolytic degradation and mechanical attributes without trade-off, extending the capability of CS as a bone graft. One such polymer is poly(ether ether ketone) (PEEK) (Fig. 1a). PEEK is a high strength, high performance aromatic thermoplastic polymer that is widely considered to cause no detrimental biological response when implanted in the body and is resistant to both hydrolytic and oxidative degradation mechanisms at temperatures far exceeding that required for medical materials within the body [24, 25]. It is utilised widely for load bearing and high-wear resistant medical devices such as spinal implants and acetabular cups [25, 26]. Availability of powdered PEEK means combination with CS powders is a viable strategy for the fabrication of reinforced composites that has not yet been reported. This work describes the production of a composite PEEK/CS material through the combination of PEEK and CS powders prior to hardening and consolidation of the PEEK phase through heating. We describe how the addition of PEEK in this manner modifies the degradation and mechanical properties of the materials.

## Methods

### Standard fabrication of PEEK/CS cylinders

Double Ground Alpha Base Crystacal (calcium sulphate hemihydrate, CSH) (CaSO_4_.0.5H_2_O) (Saint-Gobain Formula, France) and 150XF PEEK (Victrex, UK) powders were combined with 0 wt%, 2.5 wt%, 20 wt%, 40 wt% and 80 wt% PEEK by mass. Pastes of each mixture were produced under manual stirring with distilled water acquired from an arium® advance EDI pure water system (Sartorius, Germany) at a powder:liquid (P:L) ratio of 0.85 g/mL, before being poured into a mold to create cylindrical specimens of dimensions 12 mm × 6 mm. The mold was then placed upon a Denstar-500 vibrating plate (Denstar, South Korea) set at high frequency to remove air bubbles. After 10 min, the mold was transferred to an Incu-line incubator (VWR International, UK) at 37°C. After 1 h, specimens were carefully removed from the mold and heated at a ramp rate of 5 ^o^C and held at 380 ^o^C for 2.5 h in a CWF 1300 furnace (Carbolite, UK). Specimens were then stored in ambient conditions.

### Volumetric shrinkage

Cylinder volume of PEEK/CS specimens (*n* = 3) before heat treatment and following heat treatment was calculated from geometric measurements and % difference determined through calculation (Additional file 1: Equation S1).

### SEM

Secondary electron scanning electron microscopy (SEM) images of the fracture surfaces were obtained using a Sigma FE-SEM (Zeiss, Germany) operating at 15 kV under vacuum. PEEK/CS specimens with fracture surface exposed were secured firmly upon double adhesive carbon tapes attached to aluminium stubs before and gold coated under vacuum for 2 min using a K550X sputter coater (Quorum Technologies, UK) before images were acquired.

### FT-IR spectroscopy

Fourier transform infrared spectroscopy between 500 cm^−1^ and 4000 cm^−1^ was undertaken using a Nicolet 380 FT-IR spectrometer (Thermo-Scientific, USA). For sample preparation, 2 mg starting powders and powdered PEEK/CS specimens were mixed with 198 mg KBr (99.99% trace metals basis, Sigma-Aldrich, UK) and pressed into 13 mm diameter discs using a evacuable pellet die (Specac, UK) under a 10 ton force for 30 s with a hydraulic press (Specac, UK).

### Dynamic ageing protocol

Ageing was undertaken on *n* = 33 specimens per composition prepared as standard, with a further *n* = 10 specimens per composition not subjected to ageing as a control group. Individual PEEK/CS specimens were submerged in 10 mL of calcium and magnesium free Dulbecco’s phosphate buffered saline (PBS) solution (Sigma-Aldrich, UK) in screw cap 60 mL capacity clear vessels. For a fixed set of *n* = 3 specimens, a daily record of PBS supernatant pH value, wet mass, dry mass, wet height, dry height, wet mid-diameter, dry mid-diameter, wet end-diameter and dry end-diameter was recorded. Supernatant pH values were measured using a S220 SevenCompact™ pH/Ion meter (Mettler Toledo, USA) equipped with InLab Expert Pro-ISM pH electrode (Mettler Toledo, USA). PBS supernatant was drained from every specimen vessel and replenished with a further 10 mL. At 7 day intervals, *n* = 10 specimens per composition were removed from the ageing protocol until day 21.

### XRD

Powder X-ray diffraction (XRD) patterns were acquired using a D8 Autosampler Powder Diffractometer (Bruker, USA) with Cu Kα line (0.154 nm). Data was collected between 5 ^o^ and 60 ^o^ 2θ with a 0.02 ^o^ step-size and a step time of 0.5 s/^o^. Background signal was removed and intensity normalised for each scan. Patterns were matched to those stored by the International Centre for Diffraction Data (ICDD) database.

### Porosity

Apparent specimen density was calculated from geometrical measurements of *n* = 10 heat-treated PEEK/CS specimens before and during ageing. True specimen density was obtained for the same specimens using an AccuPyc II 1340 helium pycnometer (Micrometrics, USA) over 5 cycles of 5 purges. The relative density and porosity were then calculated (Additional file 1: Equation S2 and S3).

### Mechanical testing

Prior to mechanical testing, geometrical measurements of PEEK/CS specimen diameter and height were made to allow for calculations of contact area and test start height. Compression tests were undertaken on *n* = 10 specimens per composition with their long-axis perpendicular to the compression platen using a Z030 universal testing rig (Zwick/Roell, USA) equipped with a 50 kN load cell at a compression rate of 2 mm/min until specimen failure. Compressive strength values were determined by converting values of force into stress and plotting stress vs. strain curves from which the maximum value of stress corresponding to specimen failure could be obtained (Additional file 1: Equations S4 and S5, Figure S1). Compressive modulus was determined by obtaining the slope of the aforementioned curves within the elastic region (Additional file 1: Equation S6, Figure S1).

### Statistical analysis

Statistical analysis of data sets was conducted using StatPlus software. One-way ANOVA and post-hoc Turkey honest significant difference (HSD) testing was applied to compare volumetric shrinkage of specimens after heat treatment based on PEEK wt% loading. Two-way ANOVA and post-hoc Turkey HSD testing was applied to compare mechanical data of specimens based on PEEK wt% loading and ageing time. Differences were deemed significant if *p* < 0.05.

## Results and discussion

### Physical and chemical assessment of PEEK/CS cylinders

Following addition of water to starting powders a paste was formed, which hardened to form the composite material in accordance with Equation 1. Prior to heat treatment, cylindrical specimens of each composition appeared almost identical up to the point of heat treatment (Fig. 1b). Heat treatment of PEEK/CS specimens initiated melting of PEEK and dehydration of CSD (CaSO_4_.2H_2_O) to CSA (CaSO_4_) (Equation 4). These changes resulted in an alteration in both colour and apparent volume. Starting powders of both CSH (CaSO_4_.0.5H_2_O) and PEEK were both cream in colouration. Specimens containing no PEEK (0% PEEK/100%CS) remained similar in appearance after heat treatment. Specimens of 2.5%PEEKCS acquired brown speckles but still retained some cream colouration. Specimens of 20%PEEK/80%CS and 40%PEEK/60%CS became fully brown, whilst specimens of 80%PEEK/20%CS became dark brown. Although the recommended processing temperature for PEEK of 380 ^o^C was employed, polymer chain degradation can still occur leading to discoloration, which was evidently more extensive with greater loadings of PEEK (Fig. 1b).

Volumetric shrinkage of specimens also occurred as a consequence of heat treatment (Fig. 1c). Specimens with PEEK loading between 0 wt% and 40 wt% underwent a volumetric shrinkage of between approximately 9% and 15%. Specimens of 80%PEEK/20%CS however appeared significantly smaller and were confirmed to undergo the greatest extent volumetric shrinkage of 39.7 ± 0.7%. One-way ANOVA analysis confirmed that significant differences in volumetric shrinkage existed between groups based on PEEK wt% loading (F(4,10) = 87.3, *p* = 9.8x10^−8^). Post-hoc analysis utilising Turkey HSD tests found no significant differences between groups with PEEK loading between 0 wt% and 40 wt% (*p* > 0.05), however significant differences did exist between these groups and that of specimens loaded with 80 wt% PEEK (*p* < 0.001). It is recommended that fractions of CS < 40 wt% are required to maintain volumetric structure that undergoes no significant geometrical changes as a result of melting PEEK compared to unreinforced material.

**Fig. 2:**
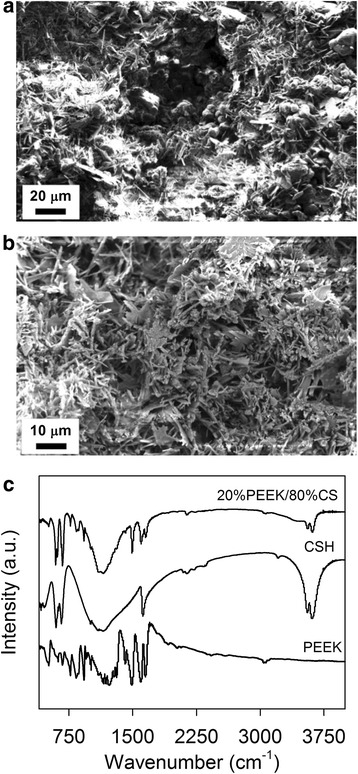
SEM micrographs of a fracture surface of a 20%PEEK/80%CS specimen prepared at a P:L ratio of 0.85 g/mL (**a**) prior to heat treatment and (**b**) after heat treatment. (**c**) FT-IR spectra between 500 cm^−1^ and 4000 cm^−1^ wavenumbers of PEEK and CSH (CaSO_4_.0.5H_2_O) starting powders, and a powdered 20%PEEK/80%CS specimen after heat treatment

Fracture surfaces of a 20%PEEK/80%CS specimen showed good dispersion of globular PEEK particles (approximately 5 μm to 10 μm) situated within a dense network of needles with high aspect ratio (approximately 20 μm by 2 μm) typical of the reported morphology for CSD (CaSO_4_.2H_2_O) (Fig. 2a). Following heat treatment, there was a change to a more flake-like morphology in addition to needles (Fig. 2b). The powdered polymeric particles were no longer apparent, since the PEEK phase had fully melted and integrated with CS crystals.

FT-IR spectra of PEEK contained peaks indicative of the chemical moieties present in its chemical structure (Figs. 1a and 2c), including aromatic and carbonyl groups [27–30]. Peaks associated with aromatic groups are located at 3072 cm^−1^ and 3034 cm^−1^ representative of = C-H stretching, and at 1592 cm^−1^ and 1486 cm^−1^ due to C = C stretching. Additional peaks at 850 cm^−1^ and 832 cm^−1^ are indicative of ring deformation vibrational modes. Peaks corresponding to carbonyl groups are found at 1652 cm^−1^, 1647 cm^−1^ and 1257 cm^−1^. Due to peak overlap at 1652cm^−1^ and 1647cm^−1^, separate bands are hard to distinguish, but indicates carbonyl stretching in amorphous and crystalline regions of PEEK respectively.

Vibrational modes of the SO_4_^2−^ anion within CSH (CaSO_4_.0.5H_2_O) were identified on the corresponding FT-IR spectrum [31–33] (Fig. 2c). A peak at approximately 1010 cm^−1^ can be assigned to SO_3_^2−^ symmetric stretching. Peaks at 1114 cm^−1^ and 1080 cm^−1^ are assignable to SO_4_^2−^ anti-symmetric stretching, whilst peaks at 658 cm^−1^ and 596 cm^−1^ are present due to SO_4_^2−^ anti-symmetric bending. Peaks relating to O-H stretching can be found between 3000 cm^−1^ and 3800 cm^−1^; peaks in the spectrum of CaSO_4_.0.5H_2_O are located at 3603 cm^−1^ and 3551 cm^−1^. A singular O-H vibrational band is also present at 1620 cm^−1^, indicative of CSH (CaSO_4_.0.5H_2_O) hydration degree.

Vibrational bands relating to both PEEK and SO_4_^2−^ anions are present in the spectrum of 20%PEEK/80%CS. The symmetric stretching peak of SO_4_^2−^ remains located at approximately 1010 cm^−1^, consistent with spectra of both CSA (CaSO_4_) and CSH (CaSO_4_.0.5H_2_O). Substantial reduction of O-H stretching peak intensity between 3000 cm^−1^ and 3800 cm^−1^ indicates dehydration of CS phase as expected (Equation 4). The singular O-H vibrational band found at 1620 cm^−1^ in the spectrum of CSH (CaSO_4_.0.5H_2_O) is no longer visible and is instead overlapped by peaks indicative of C = C stretching (1592 cm^−1^) and carbonyl stretching (1652 and 1647 cm^−1^) of PEEK polymer chains. Shaping of antisymmetric SO_4_^2−^ bending modes between 550 cm^−1^ and 750 cm^−1^, consisting of 4 overlapping bands (591 cm^−1^, 612 cm^−1^, 667 cm^−1^ and 671 cm^−1^) suggests a mixture of CSA (CaSO_4_) and CSH (CaSO_4_.0.5H_2_O). Peaks previously assigned within the FT-IR spectrum of PEEK are visible in the spectrum of 20%PEEK/80%CS including ring deformation peaks at 850 cm^−1^ and 832 cm^−1^, the C = C stretching peak at 1592 cm^−1^ and carbonyl stretching peaks 1652 cm^−1^ and 1647 cm^−1^ appeared at a relatively lower intensity, compared to in the spectrum of PEEK starting powder (Fig. 2c). Unexpectedly, this suggests PEEK is able to interact with CSA (CaSO_4_) not only physically, but also chemically through highly electron rich regions of PEEK polymer, including aromatic rings and carbonyl groups.

### Characterisation of dynamically aged PEEK/CS specimens

**Fig. 3:**
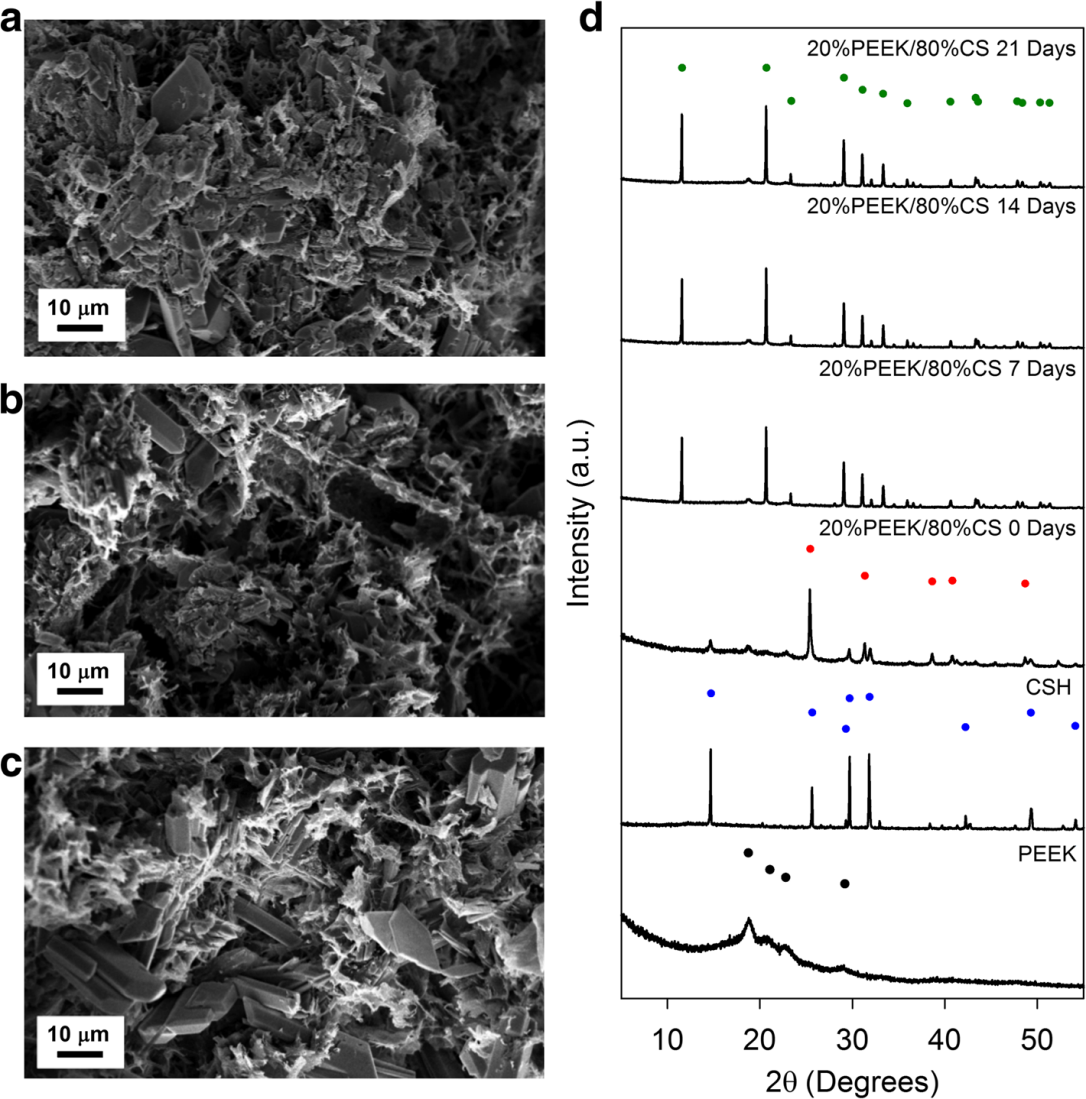
SEM images of 20%PEEK/80%CS specimens after ageing in PBS media for (**a**) 7 Days, (**b**) 14 Days and (**c**) 21 Days. (**d**) XRD diffraction patterns between 2θ values of 5 ^o^ and 60 ^o^ of PEEK and CSH (CaSO_4_.0.5H_2_O) starting powders, and powdered 20%PEEK/80%CS specimens after heat treatment prior to ageing (0 Days), and after 7 Days, 14 Days and 21 Days of ageing. An XRD pattern for PEEK from the literature confirmed the crystal structure of the polymer (•, see [30]). ICDD patterns matching CSH (CaSO_4_.0.5H_2_O) (, ICDD pattern 01-081-1448), CSA (CaSO_4_) (, ICDD pattern 01-070-0909) and CSD (CaSO_4_.2H_2_O) (, ICDD pattern 00-033-0311) (CaSO_4_.0.5H_2_O, CaSO_4_ and CaSO_4_.2H_2_O phases respectively) are also provided to aid in CS phase identification

Fracture surfaces of 20%PEEK/80%CS specimens after ageing in PBS undergo extensive microstructural transformations (Fig. 3a-c). Ageing promotes development of plate like crystal structures that are visible at Day 7 onwards, indicative of newly forming crystalline phases. This may be due to the hydrating environment provided by PBS media, promoting CS conversion from CSA (CaSO_4_) to CSD (CaSO_4_.2H_2_O) (Equation 2). Crystal structures are surrounded by a polymeric network of PEEK, distinguished by non-crystalline material interacting with crystal structures through both direct contact and coating.

Powder XRD patterns were acquired in order to assess CS phase changes as a consequence of ageing (Fig. 3d). The powder XRD pattern for PEEK consisted of broad peaks in keeping with PEEK’s semi-crystalline nature. Peaks located at 2θ values of 19 ^o^, 21 ^o^, 23 ^o^ and 29 ^o^ are in-keeping with those reported in the literature for PEEK [30]. Sharp and narrow peaks within CSH (CaSO_4_.0.5H_2_O) pattern indicated a crystalline material that matched to ICDD pattern 01-081-1448. Following heat treatment, the corresponding powder XRD pattern of a 20%PEEK/80%CS specimen contained peaks corresponding to both PEEK and CS phase components, namely CSA (CaSO_4_) and CSH (CaSO_4_.0.5H_2_O) matching ICDD patterns 01-070-0909 and 01-081-1448 respectively. After 7, 14 and 21 days of ageing, powder XRD patterns of 20%PEEK/80%CS specimens possessed peaks corresponding to CSD (CaSO_4_.2H_2_O), which were matched to ICDD pattern 00-033-0311, in addition to peaks corresponding to PEEK.

**Fig. 4:**
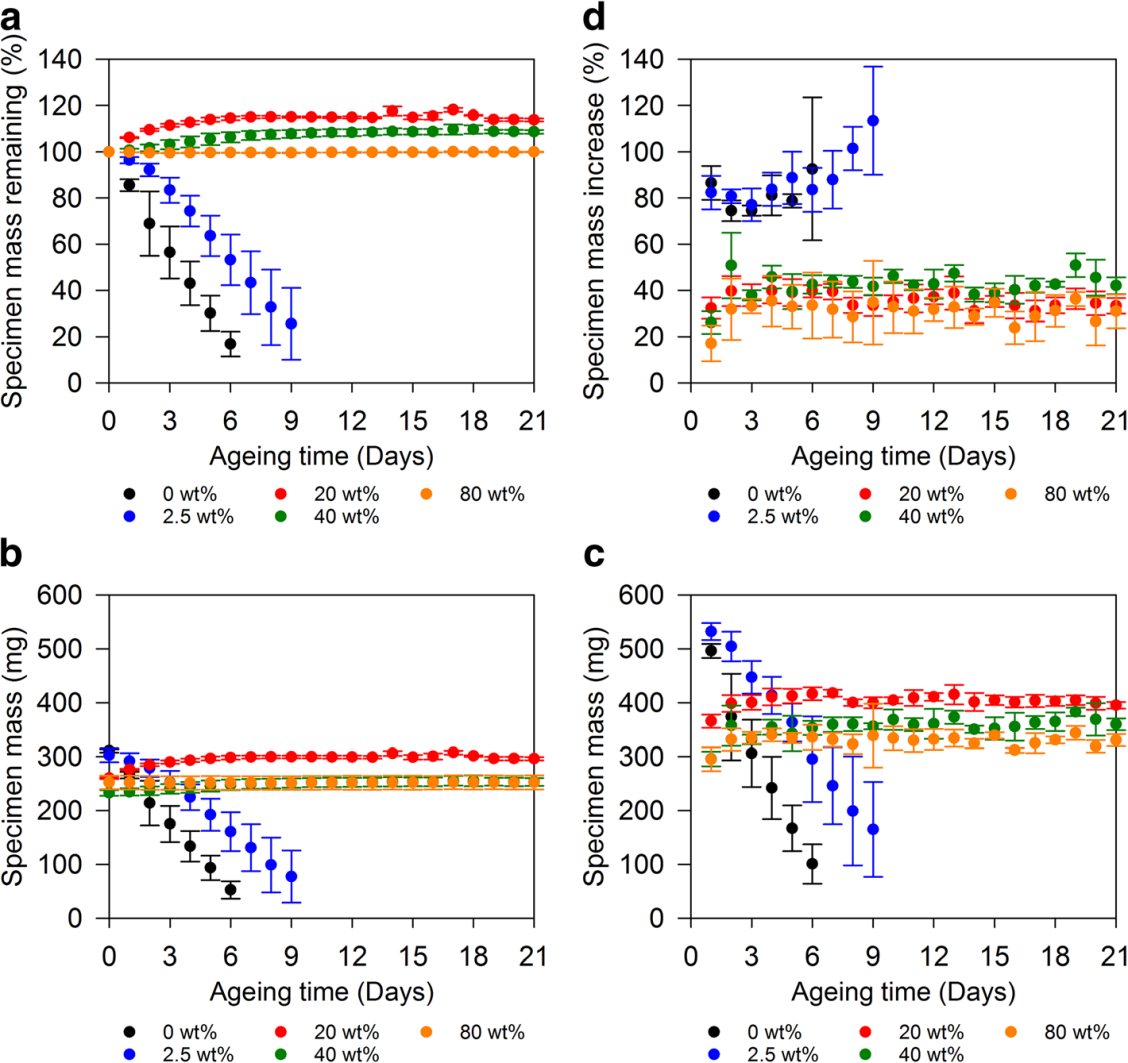
(**a**) PEEK/CS specimen mass remaining expressed as percentage (%) calculated from dry measurements. (**b**) Changes in PEEK/CS specimen mass measured dry and (**c**) wet. (**d**) PEEK/CS specimen mass increase expressed as a percentage (%) calculated between difference in dry and wet mass measurements. Error bars represent standard deviation (*n* = 3)

Submergence of heat treated PEEK/CS specimens in PBS media initiated an immediate degradation state in specimens loaded with 0% and 2.5% PEEK, however a low loading level of 2.5%, PEEK increased calcium CS longevity overall; complete deterioration of 2.5%PEEK/97.5%CS specimens did not occur until day 17 (Additional file 1: Figure S2). Mass remaining profiles calculated from dry measurements showed consistent day to day mass loss with regards to 0%PEEK/100%CS and 2.5%PEEK/97.5%CS specimens, equivalent to degradation rates of 14.1%/day of ageing (r^2^ = 0.99) and 7.9%/day of ageing (r^2^ = 0.97) respectively. This resulted in 16.9 ± 5.4% mass remaining after 6 days and 25.4 ± 15.5% mass remaining after 9 days of unreinforced PEEK/CS specimens and 2.5 wt% PEEK loaded specimens respectively (Fig. 4a). In contrast, 20%PEEK/80%CS and 40%PEEK/60%CS specimens increased in mass up to 17.6 ± 1.9% and 9.7 ± 1.8% respectively over the course of ageing duration (Fig. 4a). Deterioration of 20%PEEK/80%CS, of 40%PEEK/60%CS and 80%PEEK/20%CS specimens was not evident at any stage of ageing, due to increased physical and chemical interactions between PEEK and CS (Additional file 1: Figure S2). Interestingly, PEEK reinfrocement was shown to facilitate CS phase change from CSA (CaSO_4_) and CSH (CaSO_4_.0.5H_2_O) to CSD (CaSO_4_.2H_2_O) leading to a mass increase (Equation 1 and 2) (Figs. 3d and 4a). Theoretically, from known mass of CSA (CaSO_4_) consistuting each composite composition, calculations of maximum mass increase due to crystal hydration were 21.2% for 20%PEEK/80%CS and 15.8% for 40%PEEK/60%CS specimens, which is consistent with observed increases of 17.6 ± 1.9% and 9.7 ± 1.8% respectively. Mass changes experienced by 80%PEEK/20%CS specimens were found to be negligible, and never deviated by more than 0.5% either side of 100% of original specimen mass (Fig. 4a). Simultaneously, between 5% to 10% decreases in porosity were measured for 20%PEEK/80%CS, 40%PEEK/60%CS and 80%PEEK/20%CS specimens after 21 days ageing (Table 1), possibly arising from crystal hydration of CS and a decrease in microstructure free volume.

**Table 1:** Porosity (%) of PEEK/CS specimens after heat treatment and during ageing (*n* = 10)

Ageing time (Days)	PEEK content (%)				
	0	2.5	20	40	80
0	70.7 ± 3.15	72.1 ± 0.49	73.9 ± 1.3	73.5 ± 1.6	54.6 ± 3.4
7	-	-	65.8 ± 2.4	67.7 ± 1.4	52.0 ± 3.9
14	-	-	68.0 ± 1.0	68.2 ± 2.9	50.0 ± 2.8
21	-	-	65.9 ± 1.7	66.5 ± 1.4	49.7 ± 3.7

**Fig. 5:**
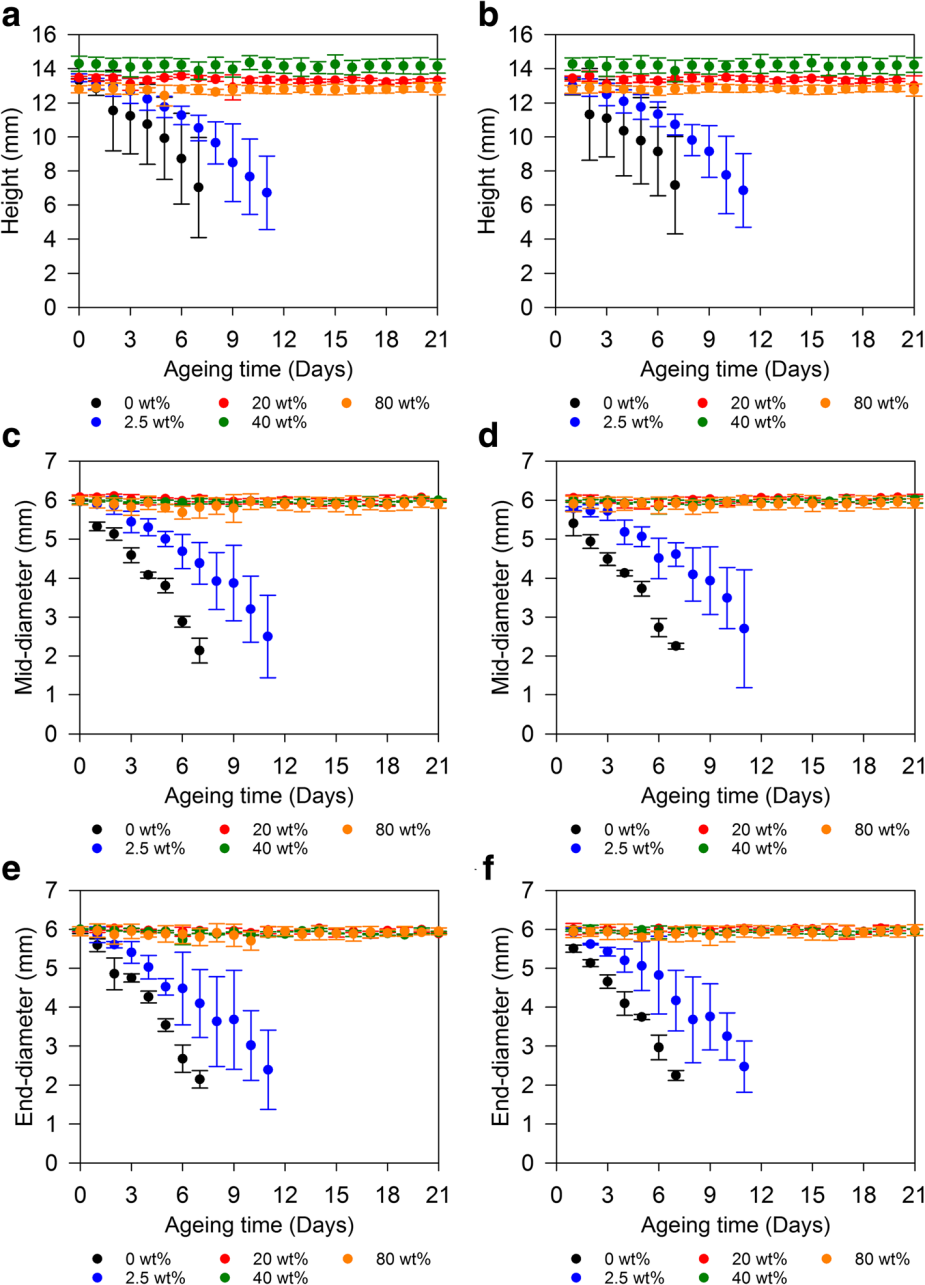
(**a**) Changes in PEEK/CS specimen height measured dry and (**b**) wet. (**c**) Changes in PEEK/CS specimen mid-diameter measured dry and (**d**) wet. (**e**) Changes in PEEK/CS specimens end-diameter measured dry and (**f**) wet. Error bars represent standard deviation (*n* = 3)

PEEK/CS specimen mass was consistently greater with regards to wet measurements compared to dry measurements (Fig. 4b,c). Specimens of 0%PEEK/100%CS and 2.5%PEEK/97.5%CS experienced daily increases in mass averaging 81.3 ± 9.4% over 6 days and 88.8 ± 10.1% over 9 days of ageing respectively (Fig. 4d). Specimens of 20%PEEK/80%CS, 40%PEEK/60%CS and 80%PEEK/20%CS experienced lower average daily increases in mass of 35.7 ± 1.3%, 42.4 ± 2.8% and 30.8 ± 3.8% respectively over 21 days of ageing (Fig. 4d). Nonetheless, this was considered as a reflection of absorptive capability and porous nature of PEEK/CS specimens, as PEEK/CS specimen height, mid-diameter and end-diameter showed no substantial differences between wet and dry measurements (Fig. 5a-f). Exploitation of absorbance capacity *in situ* may facilitate localisation of nutrients from adjacent tissues beneficial to osteoblast viability [34]. A feasible avenue of further investigation may be the loading of soluble therapeutics through liquid exchange with PEEK/CS materials to aid bone regeneration [35, 36]. Specimens of 0%PEEK/100%CS and 2.5%PEEK/97.5%CS experienced substantial losses to cylindrical dimensions in terms of height and both mid-diameter and end-diameter.

**Fig. 6:**
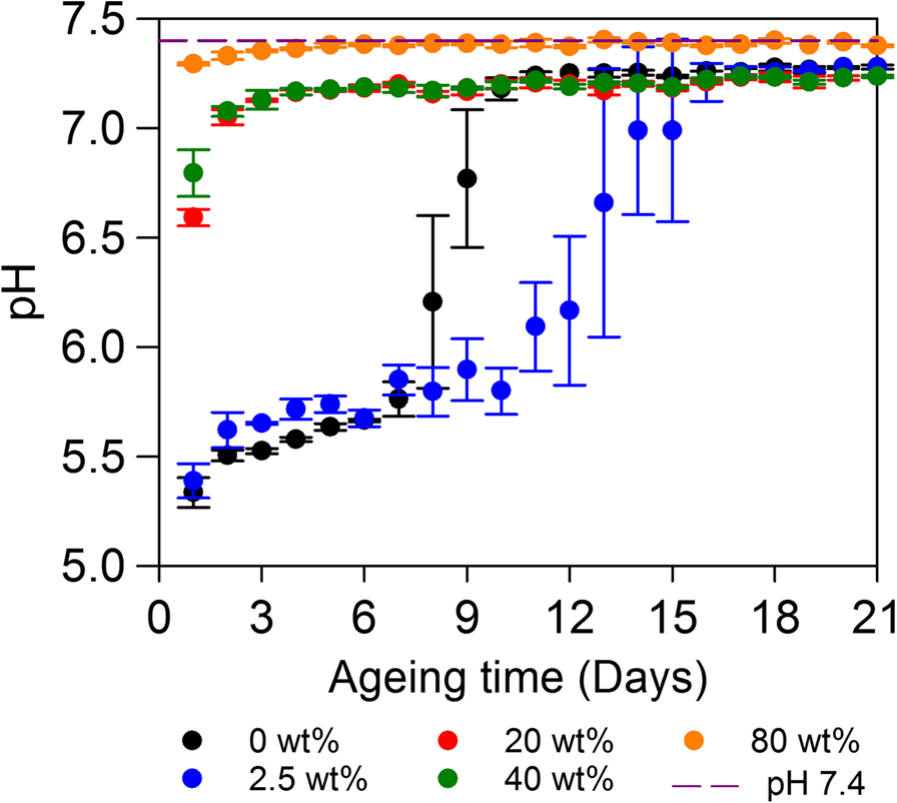
Direct pH measurements of PEEK/CS specimen PBS ageing supernatant before replenishment. PBS has a pH 7.4, and is shown in the figure by a purple dashed line as indicated by the legend. Error bars represent standard deviation (*n* = 3)

Direct measurements of PBS pH were indicative of material degradation (Fig. 6) (Additional file 1: Figure S2). Specimens of 0%PEEK/100%CS and 2.5%PEEK/97.5%CS lowered PBS pH from 7.4 to between 5 and 6. Such an environment can beneficially demineralise adjacent bone, in-turn releasing growth factors (i.e. bone morphogenic protein) that contribute to mesenchymal cell differentiation into osteoblasts, which may encourage the deposition of new bone [37, 38]. However, rapid 0%PEEK/100%CS and 2.5%PEEK/97.5%CS degradation before bone formation can occur would be a significant concern that would limit the application of these particular compositions of composite. With regards to 0%PEEK/100%CS and 2.5%PEEK/97.5%CS, the pH value of supernatant was found to decrease less extensively on a daily basis in accordance with the reducing mass of specimens due to dissolution of CS content and replenishment of PBS media (Figs. 4a-c and 6) (Additional file 1: Figure S2). Specimens of 20%PEEK/80%CS, 40%PEEK/60%CS and 80%PEEK/20%CS had little affect on pH of PBS ageing supernatant as values remained close to neutral throughout ageing (Fig. 6) (Additional file 1: Figure S2).

### Mechanical properties of PEEK/CS specimens

The compressive strength and modulus of CS (typically hydrated CSD (CaSO_4_.2H_2_O)) is typically in the range of 10 MPa to 20 MPa and between 3 GPa and 6 GPa respectively when prepared at P:L ratios between 1.5 g/mL and 2 g/mL [39, 40]. Critically, the mechanical properties of CS in this study were several orders of magnitude lower, resulting in compressive strength of 1.2 ± 0.3 MPa and compressive modulus of 0.084 ± 0.1 GPa. Preparation of pastes at P:L ratio of 0.85 g/mL (greater volume liquid fraction) evidently introduces porosity and areas of poor resistance to stress (Table 1). Further optimization of the composite may be enabled by increasing P:L ratio during PEEK/CS fabrication [41]. Furthermore, compressing specimens prior to heat treatment, and preparing pastes under vacuum may both lower the relatively high porosity of structures prior to ageing and provide additional mechanical stability during exposure to *in vitro* physiological conditions [42, 43].

**Fig. 7:**
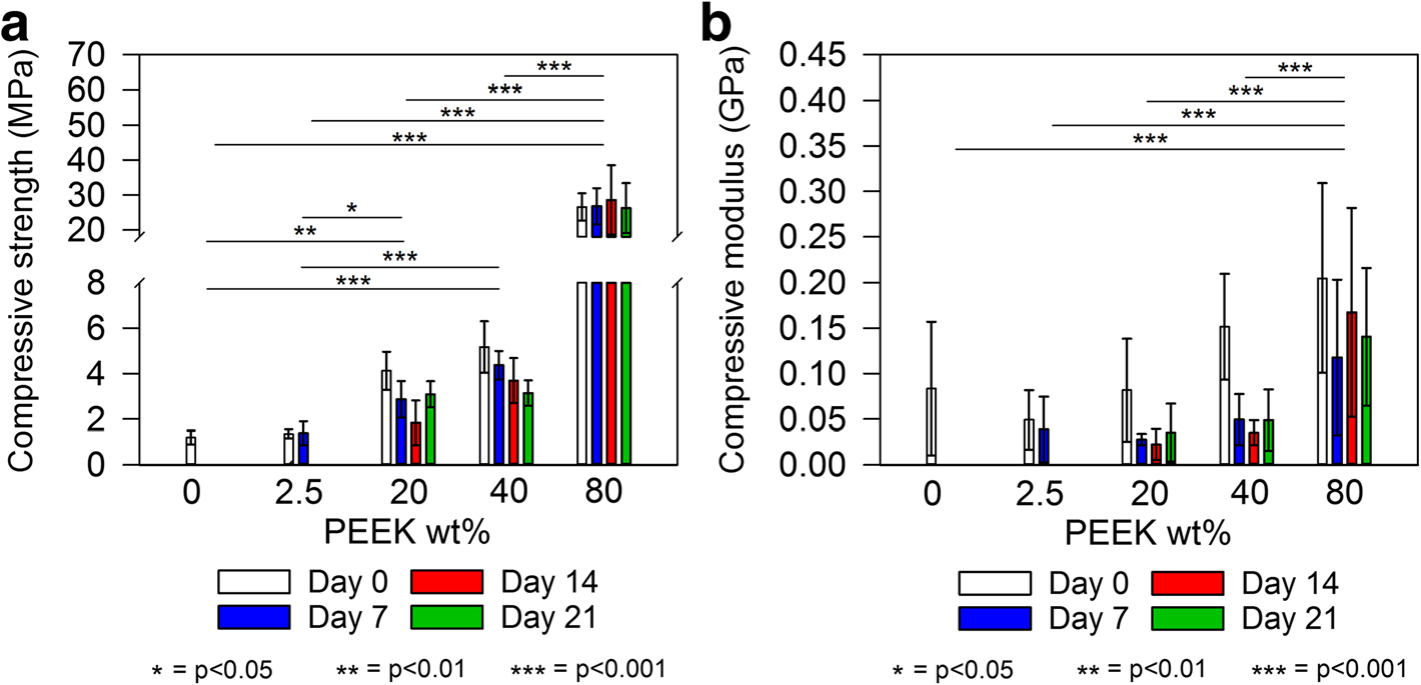
(**a**) Compressive strength and (**b**) Compressive modulus of PEEK/CS specimens after heat treatment and during ageing. Bar colouration is representative of ageing time as indicated by the legend. Error bars represent standard deviation (*n* = 10) and lines above data columns represent significant differences between groups based on PEEK wt% loading as found by post-hoc Turkey HSD tests following two-way ANOVA analysis

Importantly, our findings show PEEK in able to bring about significant gains in CS composite compressive strength based on PEEK wt% loading (Fig. 7a). Specimens of 20%PEEK/80%CS and 40%PEEK/60%CS provided approximately 3-fold and 4-fold increases in compressive strength respectively prior to ageing. Interestingly, 80 wt% PEEK loading provides a further substantial rise in compressive strength of 26.6 ± 3.9 MPa prior to ageing, equating to an approximate 22-fold gain. A significant increase was not found between groups of specimens based on ageing time (F(3,12) = 2.1, *p* = 0.16), indicating specimens did not weaken notably due to prolonged submergence in PBS media in terms of compressive strength. Promisingly, ageing did not significantly alter compressive strength of specimens over 21 days (Fig. 7a). Despite this, compressive modulus generally decreased with ageing time (Fig. 7b). Evidently, a complex relationship exists between the physical and chemical interactions of PEEK with CS, such that mechanical reinforcement is provided but not necessarily maintained during facilitation of CS phase evolution and corresponding microstructural transformations.

### Mechanism of PEEK reinforcement of CS

We propose that the strengthening and subsequent augmentation of degradation behaviour of PEEK/CS composites is due to enhanced interactions between CS crystals from the physical and chemical intercalation of PEEK following heat treatment. Without PEEK, CS is unable to retain its structure during microstructural and phase transitions initiated by PBS media. 0%PEEK/100%CS specimens likely fall apart from the infiltration of PBS solution that initiates dissolution. Heating the composites containing PEEK allows the polymer content to coat the CS crystal matrix. Even at low loadings of PEEK, such as 2.5 wt%, composites demonstrate enhanced strength and degradation response when compared to 0%PEEK/100%CS. However, the further physiochemical improvements observed at PEEK loadings of at least 20 wt% suggests that at these levels the polymer phase is able to infiltrate the entirety of structures and form a polymeric reinforcing network that binds to crystalline structures. As PEEK does not deteriorate in hydrolytic environments, the PEEK network retains structural dimensions of composites whilst protecting CS crystals from rapid deterioration in PBS media. Additionally, PEEK facilitates microstructural and compositional phase conversion of CS material from CSA (CaSO_4_) to CSD (CaSO_4_.2H_2_O) (Equation 2), which suggests that some CS crystals remain exposed to PBS. Positively therefore, bone-forming cells may still utilise the resorbable CS content available within PEEK/CS composite materials as an osteogenic scaffold for bone deposition.

## Conclusions

Acquisition of physical interactions between PEEK and CS was achieved by heating of PEEK/CS specimens. Consequentially, this led to specimen discoloration regarding specimens that contained PEEK, as well as volumetric shrinkage. Excitingly however, evidence of chemical interactions between PEEK with CS through aromatic and carbonyl moieties of the polymer chain were found to exist. The nature of these interactions significantly enhances *in vitro* dissolution behavior and physical attributes. As such, PEEK can be utilised to fundamentally improve CS attributes directly relevant to bone graft requirements, especially considering large defect volumes that require a protracted presence of osteogenic scaffold and mechanical stability.

## Additional file

Additional file 1: Supporting information for - Characterisation of a novel poly (ether ether ketone)/calcium sulphate composite for bone augmentation. File contains all additional equations and supporting figures that accompany this work. (DOCX 5127 kb)
