# Supplementary material for: Characterisation of a novel poly (ether ether ketone)/calcium sulphate composite for bone augmentation
Source: Biomater Res. 2017 May 19;21:7. doi: 10.1186/s40824-017-0093-7 (PMC5437598; doi:10.1186/s40824-017-0093-7)
Supplement: Additional file 1: — Supporting information for - Characterisation of a novel poly (ether ether ketone)/calcium sulphate composite for bone augmentation. File contains all additional equations and supporting figures that accompany this work. (DOCX 5127 kb) [file 40824_2017_93_MOESM1_ESM.docx]

# Additional file 1

**Supporting information for - Characterisation of a novel poly(ether ether ketone)/calcium sulphate composite for bone augmentation**

Erik A. B. Hughes^1^ and Liam M. Grover^1^*

^1^ School of Chemical Engineering, University of Birmingham, Edgbaston, B15 2TT, UK

* Corresponding author - L.M.Grover@bham.ac.uk

Equations used in this work:

% volumetric shrinkage = ((V_1_ – V_2_) / V_1_) x 100 (Equation S1)

Where,

V_1_ = Volume of specimen prior to heat treatment (mm^3^)

V_2_ = Volume of specimen after heat treatment (mm^3^)

ρ_rel_ = ρ_app_ / ρ_true_ (Equation S2)

Where,

ρ_rel_ = Relative density of specimen (g/cm^3^)

ρ_app_ = Apparent density of specimen (g/cm^3^)

ρ_true_ = True density of specimen (g/cm^3^)

Porosity = (1 - ρ_relative_) x 100 (Equation S3)

Where,

ρ_relative_ = Relative density of specimen

σ = F / A (Equation S4)

Where,

σ_comp_ = Compressive stress (MPa)

F = Loading force (N)

A = Specimen contact area (mm^2^)

ε = l_change_ / l_original_ (Equation S5)

Where,

ε = Strain

l_change_ = Change in specimen height (mm)

l_original_ = Original specimen height (mm)

E_comp_ = (σ / ε) / 1000 (Equation S6)

Where,

E_comp_ = Compressive modulus (GPa)

σ = Stress (MPa)

ε = Strain


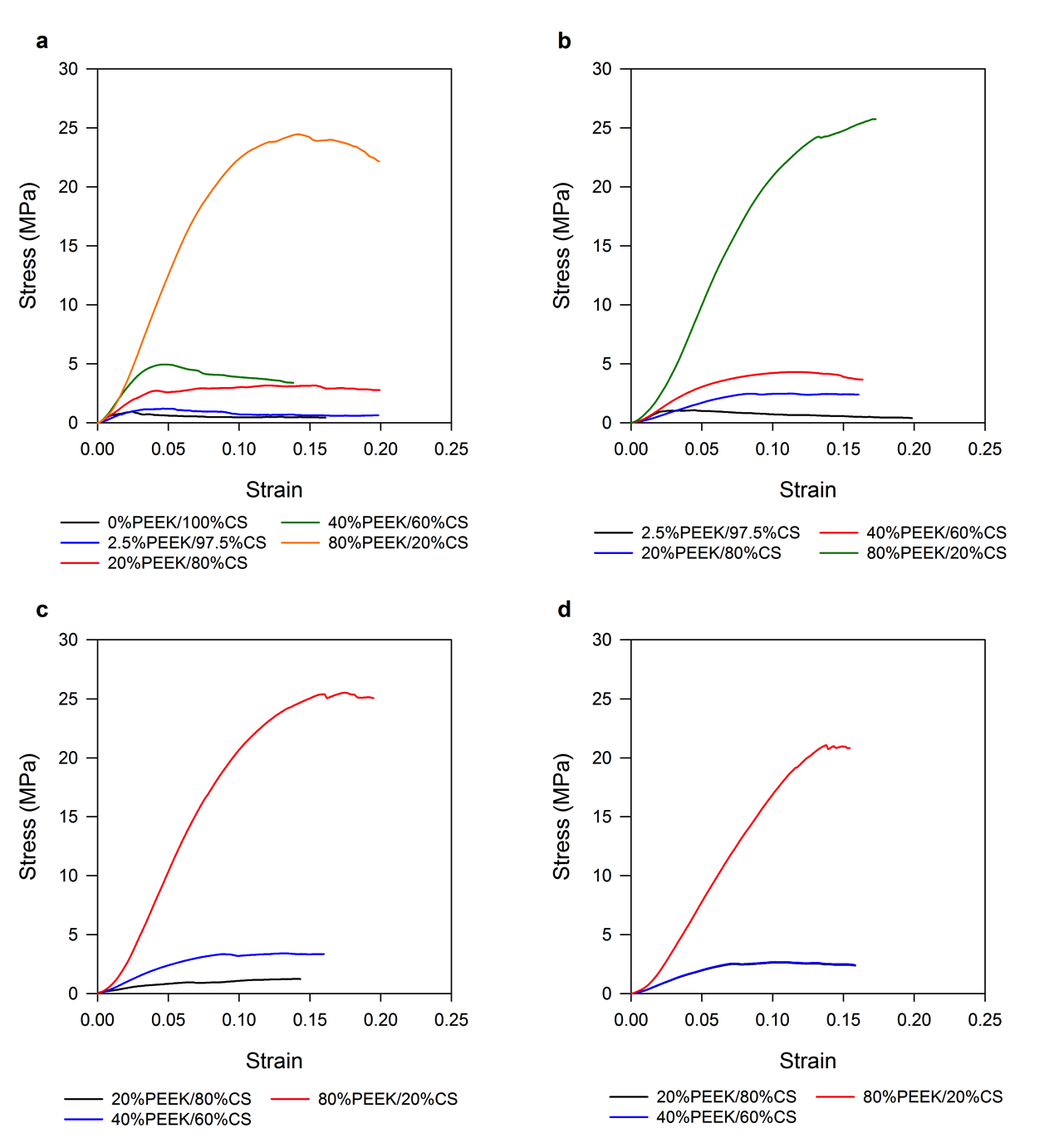


**Figure S1.** Averaged stress vs. strain curves for n=10 PEEK/CS specimens tested **(a)** after heat treatment prior to ageing (0 Days) and **(b)** after 7 Days, **(c)** 14 Days and **(d)** 21 Days of ageing.

**
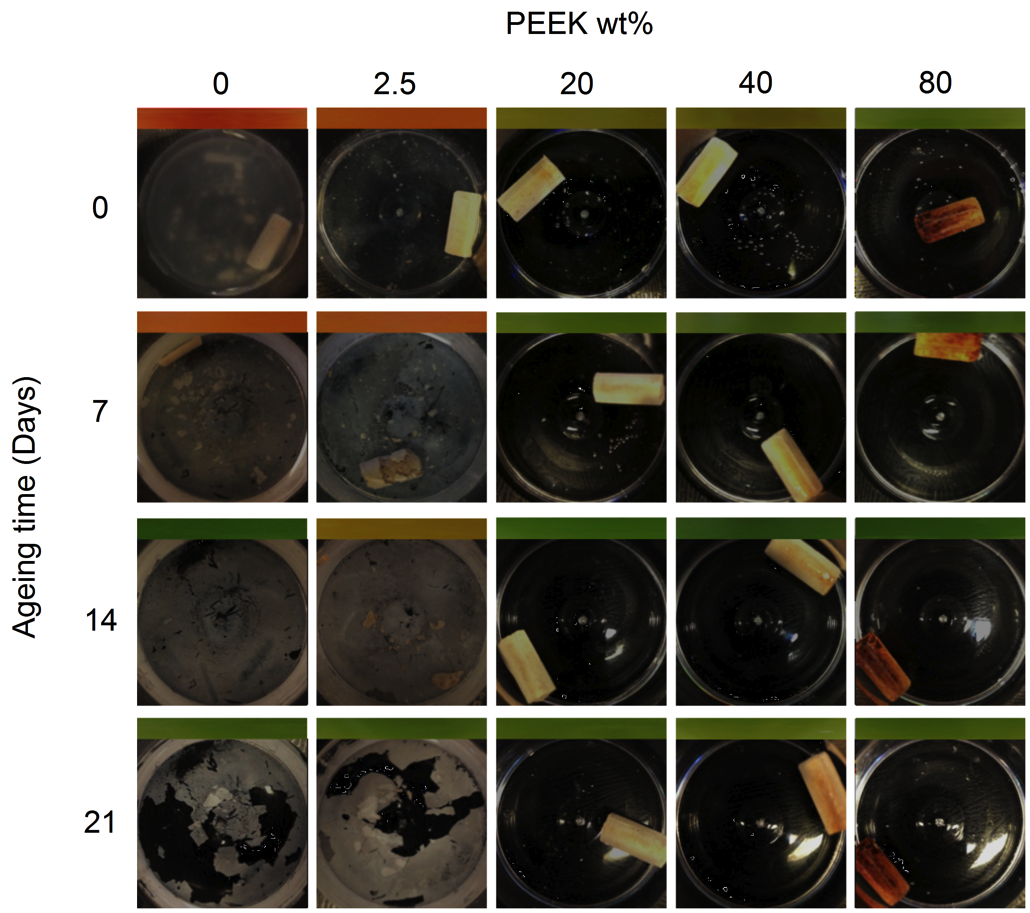
**

**Figure S2.** Top down view of PEEK/CS specimens during dynamic ageing study at 1 day, 7 days, 14 days and 21 days, bar above individual images is the colouration of an aliquot of PBS ageing media after addition of universal pH indicator before replenishment (PBS media was green) (orange = pH 5 – 5.5, yellow = pH 6 – 6.5, green = pH 7 - 7.5).
